# Down-Regulation of Cytokinin Oxidase 2 Expression Increases Tiller Number and Improves Rice Yield

**DOI:** 10.1186/s12284-015-0070-5

**Published:** 2015-12-07

**Authors:** Su-Ying Yeh, Hau-Wen Chen, Chun-Yeung Ng, Chu-Yin Lin, Tung-Hai Tseng, Wen-Hsiung Li, Maurice S. B. Ku

**Affiliations:** Biodiversity Research Center and Genome Research Center, Academia Sinica, Taipei, 115 Taiwan; Department of Bioagricultural Science, National Chiayi University, Chiayi, 600 Taiwan; Division of Biotechnology, Taiwan Agricultural Research Institute, Taichung, 413 Taiwan; Department of Ecology and Evolution, University of Chicago, Chicago, IL 60637 USA; School of Biological Sciences, Washington State University, Pullman, WA 99164 USA

**Keywords:** Rice, Cytokinin oxidase/dehydrogenase, shRNA, Growth, Productivity

## Abstract

**Background:**

Cytokinins are plant-specific hormones that affect plant growth and development. The endogenous level of cytokinins in plant cells is regulated in part by irreversible degradation via cytokinin oxidase/dehydrogenase (CKX). Among the 11 rice CKXs, CKX2 has been implicated in regulation of rice grain yield.

**Results:**

To specifically down-regulate *OsCKX2* expression, we have chosen two conserved glycosylation regions of *OsCKX2* for designing artificial short hairpin RNA interference genes (shRNA-*CX3* and -*CX5*, representing the 5′ and 3′ glycosylation region sequences, respectively) for transformation by the *Agrobacterium*-mediated method. For each construct, 5 independent transgenic lines were obtained for detailed analysis. Southern blot analysis confirmed the integration of the shRNA genes into the rice genome, and quantitative real time RT-PCR and northern blot analyses showed reduced *OsCKX2* expression in the young stem of transgenic rice at varying degrees. However, the expression of other rice *CKX* genes, such as *CKX1* and *CKX3*, in these transgenic lines was not altered. Transgenic rice plants grown in the greenhouse were greener and more vigorous with delayed senescence, compared to the wild type. In field experiments, both *CX3* and *CX5* transgenic rice plants produced more tillers (27–81 %) and grains (24–67 %) per plant and had a heavier 1000 grain weight (5–15 %) than the wild type. The increases in grain yield were highly correlated with increased tiller numbers. Consistently, insertional activation of *OsCKX2* led to increased expression of *CKX2* and reduced tiller number and growth in a gene-dosage dependant manner.

**Conclusions:**

Taken together, these results demonstrate that specific suppression of *OsCKX2* expression through shRNA-mediated gene silencing leads to enhanced growth and productivity in rice by increasing tiller number and grain weight.

**Electronic supplementary material:**

The online version of this article (doi:10.1186/s12284-015-0070-5) contains supplementary material, which is available to authorized users.

## Background

Cytokinins are a class of plant hormones that play an essential role in various developmental and physiological processes, including cell differentiation, apical dominance, leaf senescence, nutrient signaling, and shoot and chloroplast differentiation (Mok 1994; Letham 1994). One of the mechanisms that controls the endogenous level of cytokinins is mediated by the enzyme cytokinin oxidase/dehydrogenase (CKX) (Galuszka et al. 2001), which irreversibly degrades cytokinins in plants (Jones and Schreiber 1997). Genes coding for CKXs have been cloned and characterized from model plants, such as *Arabidopsis* (Werner et al. 2003; Werner et al. 2001) and rice (Ashikari et al. 2005). The enzyme is encoded by a multigene family, including 7 in *Arabidopsis* (*AtCKX*) and 11 in rice (*OsCKX*). The CKX proteins encoded by the *Arabidopsis* gene family vary in their biochemical properties, especially in substrate specificity (Galuszka et al. 2007). Tsai et al. (2012) suggested that each rice *CKX* gene has a distinct role in the regulation of the cytokinin level in different tissues, because CKXs exhibit differential expression and regulation patterns in roots and shoots.

CKX proteins are widely distributed in plants (Schmulling et al. 2003) and are generally classified as glycoproteins (Armstrong 1994). Synthesis and accumulation of CKXs are subjected to multiple regulatory mechanisms, depending on endogenous cytokinin contents in plant cells (Kowalska et al. 2010). Interestingly, expression of multiple *CKX* genes is differentially regulated in roots and shoots of rice in response to treatment with exogenous cytokinin (Tsai et al. 2012). For example, *OsCKX2* transcript increases in rice shoots, but the expression of *OsCKX6* is down-regulated specifically in the shoots in response to cytokinin treatment (Tsai et al. 2012). CKXs are glycosylated posttranslationally. Glycosylation, the attachment of sugar moieties to proteins, is one of the important post-translational modifications in eukaryotic protein biosynthesis (Varki et al. 1995). Glycosylation of proteins in plants affects protein structure, folding, stability and biological activity (Ceriotti et al. 1998; Severino et al. 2010). It has been demonstrated in tobacco that the cytokinin-induced upregulation of CKX activity is mainly associated with the *N*-glycosylated form of the enzyme, suggesting that the control of CKX activity is at least in part dependent on glycosylation (Motyka et al. 2003). Despite our understanding of the biochemical properties of CKXs, the specific function and regulation of CKXs in plants have not been fully elucidated.

Most agriculturally important traits in crops, such as yield and quality, are controlled by several genes known as quantitative trait loci (QTLs) (Collard et al. 2005). In the past decade, efforts have been made to characterize QTLs for grain productivity. QTL analysis in rice has suggested that a locus responsible for grain yield, *Gn1a*, encodes cytokinin oxidase/dehydrogenase (OsCKX2) (Ashikari et al. 2005). Decreased expression of *OsCKX2* in transgenic rice harboring antisense *OsCKX2* cDNA resulted in increased grain number in the panicle (Ashikari et al. 2005). CKX is therefore regarded as a negative regulator of cytokinin metabolism. Consistent with this view, high-yielding rice cultivars which produce more grains in the panicles were found to have reduced or lost function of *OsCKX2* and had higher cytokinins accumulated in the inflorescence meristems. In good agreement, a double mutant of *CKX3* and *CKX5* in *Arabidopsis* formed more and larger flowers (Bartrina et al. 2011). Thus, there is an increasing interest in manipulating *CKX* gene expression in crops as a means to alter their growth and development through enhanced or decreased cytokinin levels. An earlier study showed that overexpression of *AtCKX* gene in transgenic tobacco plants led to reduced endogenous cytokinin contents (Werner et al. 2001). Suppression or overexpression of *GhCKX* (*Gossypium hirsutum* L.) in transgenic tobacco gave rise to a cytokinin-overproducing (e.g., more flowers and capsules) or cytokinin-deficient (e.g., fewer or no flowers) phenotype (Zeng et al. 2012). In addition, silencing of *HvCKX1*-hpRNAi (hairpin RNA interference) in transgenic barley resulted in a lower CKX activity and a higher grain yield (Zalewski et al. 2010). Taken together, these results suggest that crop yields can be manipulated by altering the expression of *CKX* genes and cytokinin contents.

Rice (*Oryza sativa* L.) is one of the most important crops and food sources worldwide. To study the role of CKX2 in relation to rice productivity, we specifically down-regulated the expression of *OsCKX*2 by RNAi in this study, using stable transformation of short hairpin RNAs (shRNAs) that comprised the *N*-glycosylation site sequences of *OsCKX2* (Brummelkamp et al. 2002). Our results showed that these shRNAs efficiently and specifically suppress *OsCKX2* expression in transgenic rice, leading to enhanced growth and productivity via increased tiller numbers and grain weight. Consistently, insertional activation of *OsCKX2* in transgenic rice resulted in increased expression of *OsCKX2* with the plants showing a decreased tiller number phenotype.

## Results

### PCR and RT-PCR Analyses of Primary To Transformants

**Fig. 1:**
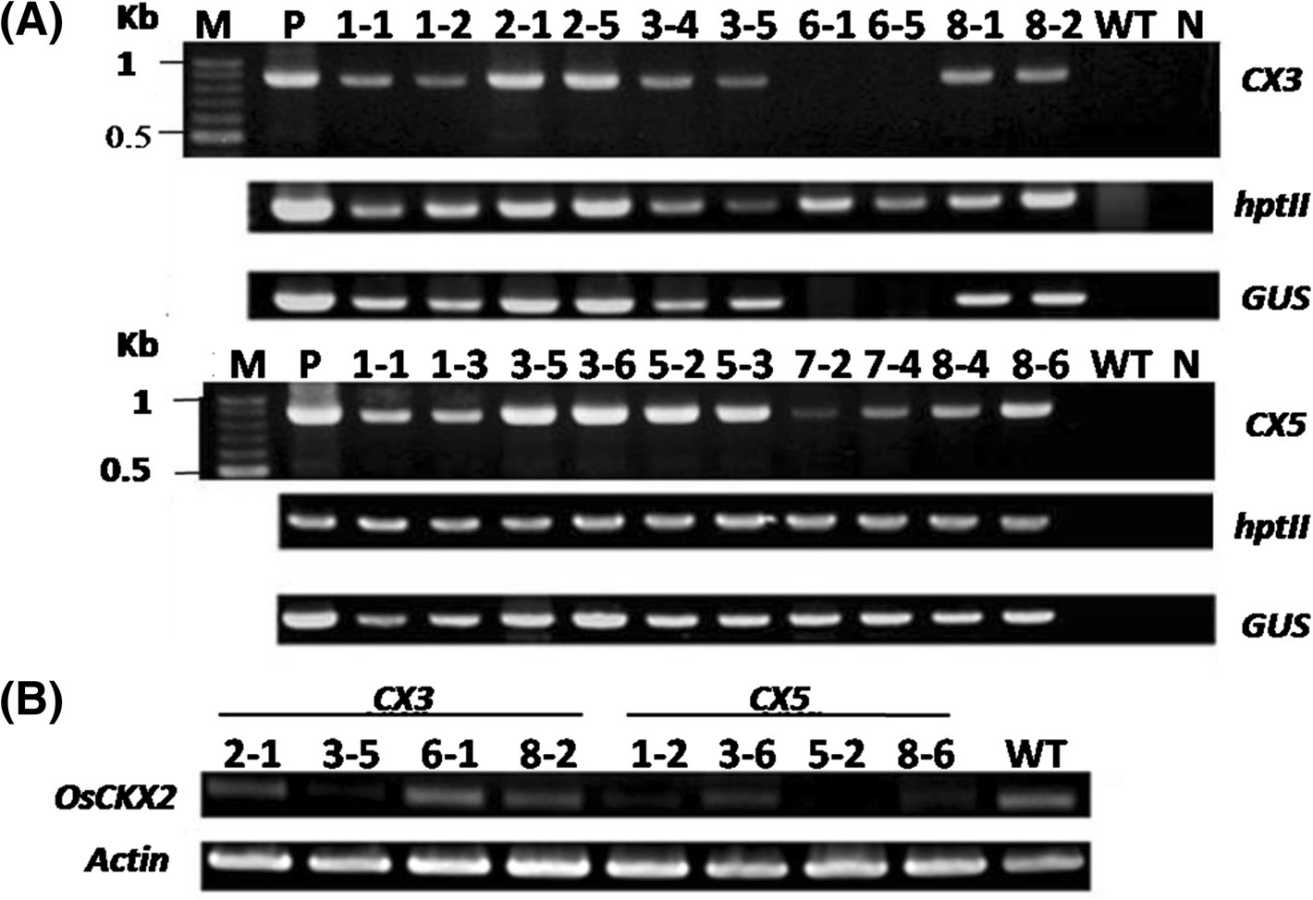
Molecular analysis of selected primary *OsCKX2* transformants (T0) and wild-type (WT) plants. **a** Detection of transgenes in selected primary transformants. The presence of *CX3* (or *CX5*), *hptII* and *GUS* from selected primary transformants was detected by PCR. M: DNA markers, P: positive control (plasmid DNA as template), WT: untransformed wild type, N: negative control (H_2_O as template). **b** Expression of *OsCKX2* in selected primary transformants and wild type (WT), as assayed by RT-PCR using specific primers. Expression of *actin* was used as a cDNA loading control

For each transformation vector, a total of 10 primary T0 transformants from five independent transgenic lines were first screened for the presence of *CX3* or *CX5* transgene by PCR. Eight individual transformants from the *CX3*- and ten from the *CX5*-suppression rice lines were detected positive for *hptII*, *GUS* and *CX3* (or *CX5*) genes (Fig. 1a). These transgenes were not detected in the wild type (WT) or negative control (H_2_O as template). Moreover, the PCR products for these transgenes were sequenced, showing the correct sequences (data not shown). Representative T0 transgenic lines containing all of these three transgenes were selected for further analysis.

The expression of *OsCKX2* in the leaves of selected primary transformants was first analyzed by semi-quantitative RT-PCR (RT-PCR) using specific primers. There was variation in the expression level of *OsCKX2* among the selected transformants. In general, the *OsCKX2* transcript levels were much lower in the transformants than in WT (Fig. 1b), except for line 6–1 of the *CX3*-suppression line (Fig. 1a). The *OsCKX2* transcript level in line 6–1 was the same as that in the control, which could be attributed to the low expression of shRNA-*CX3* in this transgenic line. Overall, the results showed that shRNA-*CX3* and -*CX5* introduced into the rice genome resulted in reduced expression of *OsCKX2* in these transgenic lines.

### Southern Analysis of To Transgenic Rice

**Fig. 2:**
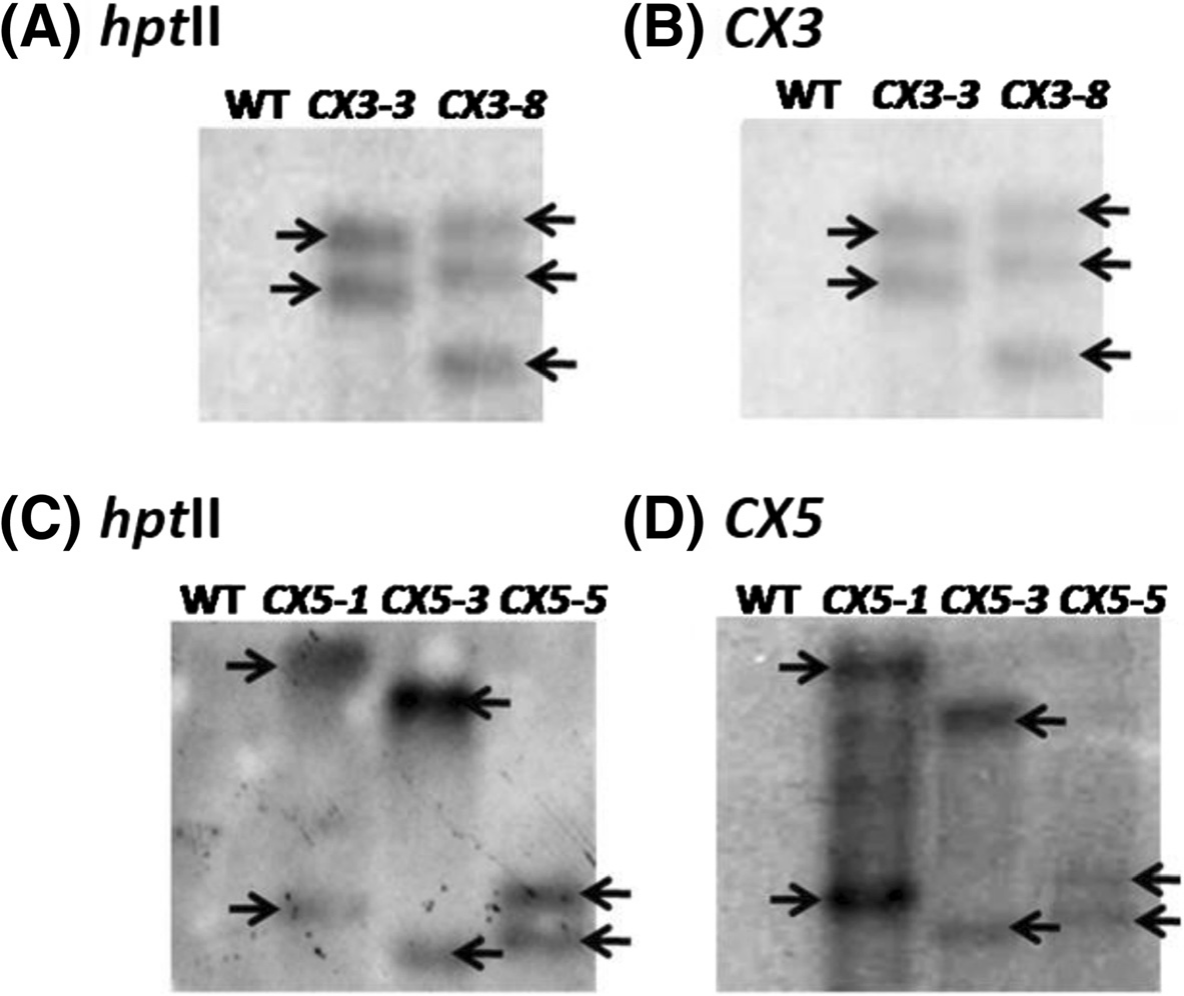
Southern blot analysis of *hptII*, *CX3* and *CX5* in selected T0 primary rice transformants. The T0 primary transformants selected included two independent transgenic lines (*CX3-3* and *CX3-8*) from transformation with shRNA-*CX3* (**a**, **b**) and three independent transgenic lines (*CX5-1*, *CX5-3* and *CX5-5*) from transformation with shRNA-*CX5* (**c**, **d**). WT: untransformed wild type. Genomic DNA was digested with *Sac*I (**a**, **b**) or *Hin*dIII (**c**, **d**) and hybridized with a ^32^P-labelled probe corresponding to *hptII* (**a**, **c**), *CX3* (**b**), or *CX5* (**d**). The number of reactive bands in each lane represents the transgene copies in each transgenic line

Based on the preliminary results of PCR and RT-PCR analyses, representative T0 transgenic lines were selected for gene integration analysis. Southern blot analysis indicated that both *hptII* and *CX3* genes on the T-DNA were integrated into the rice genome at 2 and 3 copies in the *CX3-3* and the *CX3-8* transgenic lines, respectively (Fig. 2a, b). Two copies of the *hptII* and *CX5* genes were present in the *CX5-1*, *CX5-3*, and *CX5-5* transgenic lines (Fig. 2c, d). The same pattern of integration of both *hptII* and *CX3* (or *CX5*) in the rice genome indicates that the two transgenes on the T-DNA were transferred together.

### *OsCKX2* Expression Profile and Silencing of *OsCKX2* Expression in Transgenic Rice

**Fig. 3:**
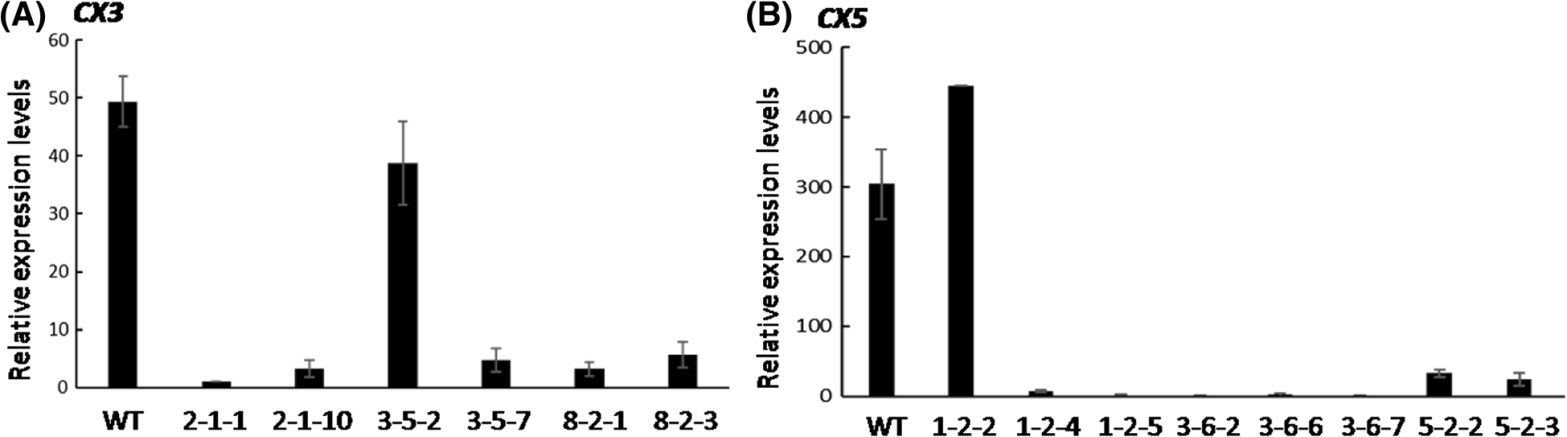
Relative *OsCKX2* expression levels in selected T1 *CX3*- and *CX5*-suppression lines by qRT-PCR. Total RNA was isolated from young stems of wild type (WT) and selected T1 *CX3*-suppression lines (2–1–1, 2–1–10, 3–5–2, 3–5–7, 8–2–1, 8–2–3) (**a**) and *CX5*-suppression lines (1–2–2, 1–2–4, 1–2–5, 3–6–2, 3–6–6, 3–6–7, 5–2–2, 5–2–3) (**b**). *17S rRNA* was used as an internal control for normalization. The expression level of line 2–1–1 and line 3–6–7 were used as a reference in the comparison of *CX3-* and *CX5*-suppression lines, respectively. Values presented were mean +/− SD of 3 replicates of cDNA samples

The expression profiles of *OsCKX2* in different organs (leaf, root, stem, inflorescence and flower) of WT plants were examined by RT-PCR first. As shown in Additional file 1: Figure S1A, low transcript levels of *OsCKX2* were detected in leaves and roots, but a high transcript level was detected in the stems of the WT plants. In contrast, the representative transformants of *CX3*- and *CX5*-suppression transgenic lines showed a significantly reduced *OsCKX2* transcript level in the young stems, as compared to WT (Additional file 1: Figure S1B). Next, the transcript levels of *OsCKX2* in the young stems of different transgenic lines were evaluated by northern gel blot analysis, relative to that of WT. The representative plants of selected independent transgenic lines all exhibited low levels of *OsCKX2* transcript (Additional file 1: Figure S1C). The expression of *OsCKX2* in the *CX3*- and *CX5*-supression transgenic lines was further analyzed by quantitative real time PCR (qRT-PCR). The results indicated that both constructs effectively suppressed the expression of *OsCKX2* in most T0 plants (Fig. 1b), but shRNA-*CX5* construct was more effective in suppressing its expression than shRNA-*CX3* construct (Fig. 3).

To test whether the RNAi-*OsCKX2* constructs also down-regulated the expression of other *OsCKX* genes, the expression of *CKX1* and *CKX3* in the *CX3*- and *CX5*-suppression lines was examined by qRT-PCR. Phylogenetic analysis (Matsuo et al. 2012) showed that rice *CKX1* is most closely related to *OsCKX2* (51.7 % identity in amino acid sequence). Rice *CKX3* (*OsCKX3*) was randomly selected among other *OsCKX* genes (Matsuo et al. 2012; Li et al. 2013). Our results revealed that expression of both rice *CKX1* or *CKX3* was not significantly altered in both suppression lines (Additional file 2: Figure S2). These results suggest that the integration of shRNA-*CX3* or -*CX5* into the genome of these transgenic rice plants has caused sequence-specific interference with homologous *OsCKX2* expression withouting affecting the expression of other *CKX* genes.

### Phenotypic Characterization of *CX3*- or *CX5*-Suppression Transgenic Rice

**Fig. 4:**
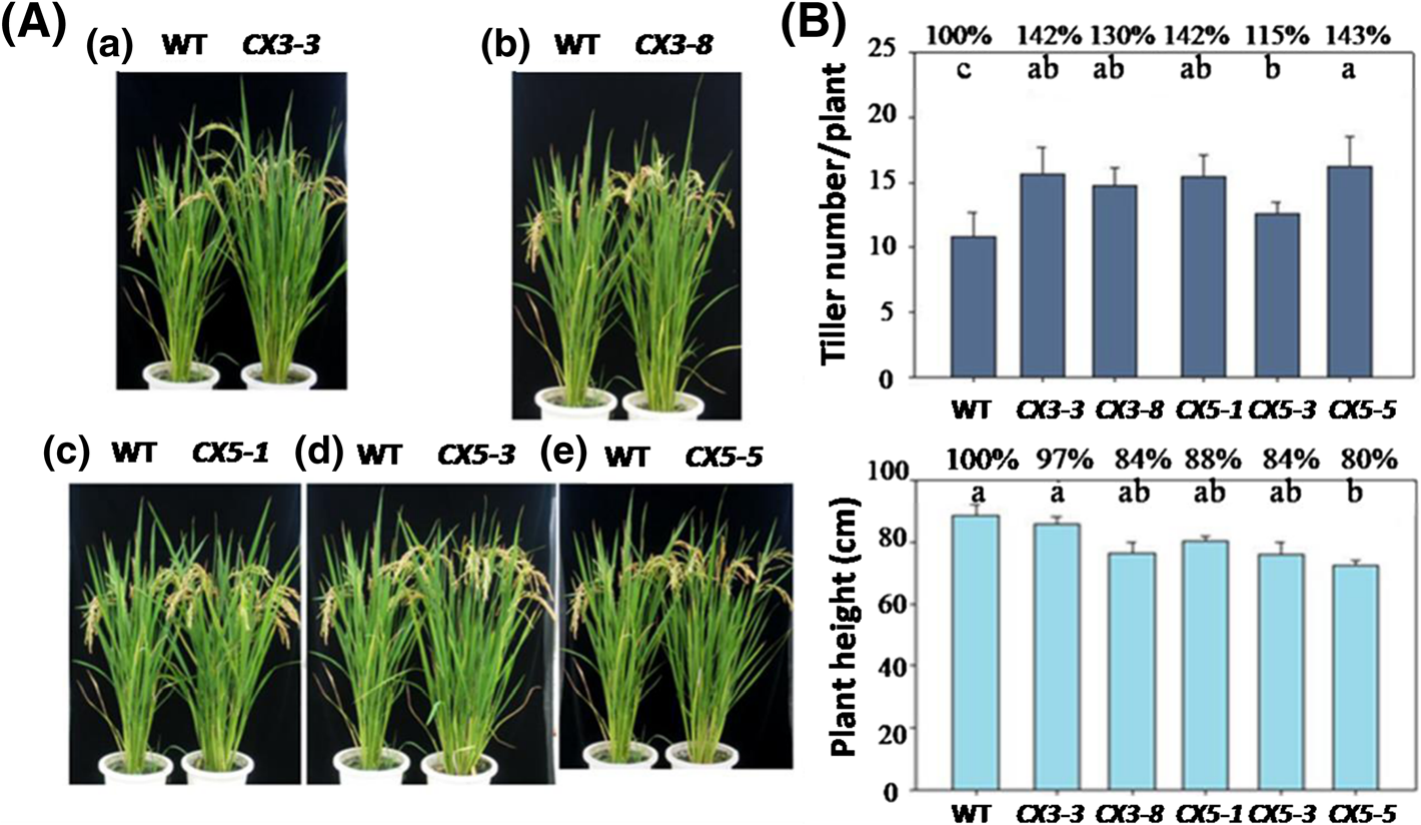
Growth phenotypes of representative T3 *CX3*- and *CX5*-Suppression transgenic rice lines grown in the greenhouse. **a** Mature plants of 4-month-old untransformed wild type (WT) and selected transgenic lines (lines 3 and 8 from transformation with *p*shRNA-*CX3*; lines 1, 3 and 5 from transformation with *p*shRNA-*CX5*). **b** Tiller number/plant and plant height analyzed upon harvest. The % data presented are expressed relative to the control (100 %). Values are mean ± SD (*n* = 7), *P* <0.01. WT: untransformed wild-type plants; *CX3* suppression lines (*CX3-3* and *CX3-8*); *CX5* suppression lines (*CX5-1*, *CX5-3* and *CX5-5*)

For growth analysis, attempts were made first to screen homozygous suppression lines based on hygromycin resistance during seed germination since *hptII* and *CX3* (or *CX5*) were integrated together (Fig. 2). About 80 self-pollinated T1 seeds from selected T0 lines were germinated on 1/2 MS medium containing 50 mg L^−1^ hygromycin. For each line, vigorous seedlings from germinated seeds were selected randomly to grow in pots, and self-pollinated T2 seeds were subjected to another round of screening. The T3 seeds from two *CX3* and three *CX5* T2 lines showed 100 % germination on hygromycin-containing MS medium, which were considered homozygous for the introduced genes. Since both *CX3* and *CX5* transgenic lines contained 2–3 copies of the transgenes based on Southern analysis (Fig. 2), the possibility that some of the T3 transgenic lines could be heterozygous for some of the transgenes introduced cannot be totally ruled out. Hygromycin resistant T3 seedlings were transferred to soil and grown in the greenhouse for phenotype observation. The phenotypes of *CX3-* and *CX5-*suppression rice plants were compared with that of the WT at different developmental stages. All transgenic plants appeared uniformly greener than WT at all stages and showed a delayed senescence. For example, three-month-old transgenic plants had a higher leaf chlorophyll content (3–9 %, significance level at *P* <0.05) for two of the five lines (data not shown). Morphological comparison showed that all transgenic lines produced more tillers with a high fertility (four-month-old plants shown in Fig. 4a). In comparison to WT, the number of mature tillers increased by 15–43 %, while the plant height was reduced by 3–12 % in the T3 transgenic rice (Fig. 4b).

### Agronomic Traits of T3 Transgenic Rice in the Field

**Fig. 5:**
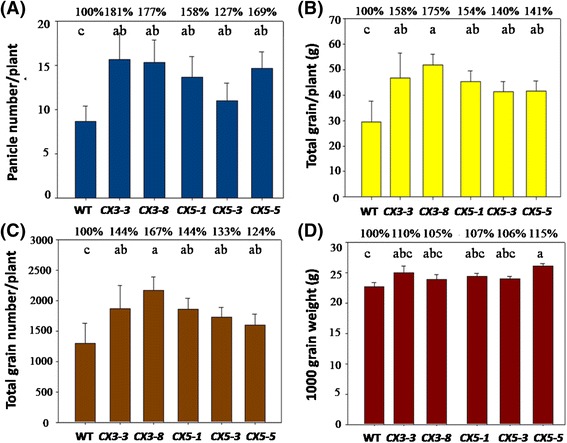
Agronomic traits of wild-type (WT) and T3 *OsCKX2* suppression lines grown in the field. **a** Panicle number/plant, (**b**) total grain weight/plant, (**c**) total grain number/plant and (**d**) 1000 grain weight. Abbreviations for transgenic lines are the same as described in Fig. 5. Values are mean ± SD (eight replicates with 5 plants/replicate); different letters denoted significance level at *P* <0.01. The % data presented are expressed relative to the control (100 %)

To assess the influence of decreased expression of *OsCKX2* on growth and productivity, homozygous T3 seedlings of two *CX3* and three *CX5* suppression rice lines were grown in the field with one seedling per hill. At maturity, the panicle number per plant increased significantly, by 27–81 %, in these transgenic rice lines, as compared to WT (Fig. 5a). *CX3-3* and *CX3-8* suppression lines also exhibited an increase in total grain number/plant, by 44 and 67 %, and in total grain weight/plant, by 58 and 75 %, respectively (Fig. 5b, c). Consistently, suppression lines *CX5-1*, *CX5-3* and *CX5-5* also showed an increase in total grain number/plant, by 54, 40 and 41 %, and in total grain weight/plant, by 44, 33 and 24 %, respectively, relative to WT (Fig. 5b, c). One thousand grain weight (g) also increased by 5–15 % in these transgenic rice lines (Fig. 5d). The enhancements in these agronomic traits were significant at *P* <0.01. Taken together, these results suggest that transgenic rice carrying the *CX3*- or *CX5*-silencing gene exhibit significant promotion in growth, leading to increased grain productivity, mainly due to a combination of increased panicle number and heavier grain weight.

### Growth Traits of Transgenic Rice with Insertional Activation of *CKX2*

**Fig. 6:**
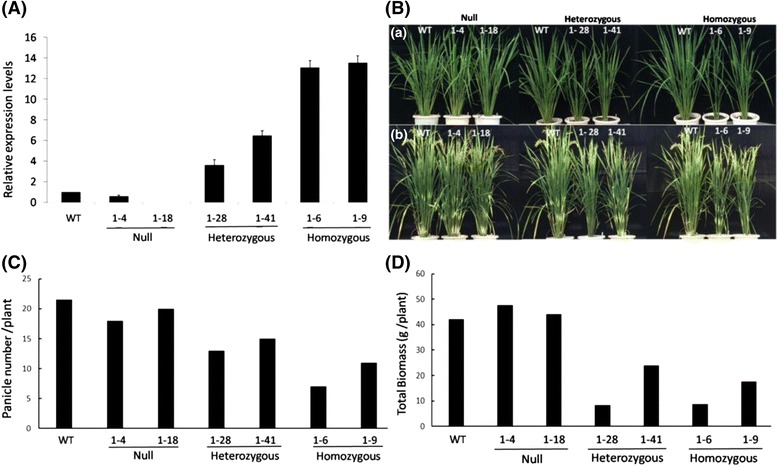
Relative expression levels of *OsCKX2* (**a**) and growth traits (**b**, **c**, **d**) of representative null, heterozygous and homozygous T1 plants derived from *OsCKX2*-overexpression line, as analyzed by qRT-PCR. **a** Total RNA was isolated from the leaves of wild type (WT), null lines (1–4, 1–18), *CKX2*-overexpression heterozygous (1–28, 1–41) and homozygous (1–6, 1–9) lines. *17S rRNA* was used as an internal control for normalization. The expression level of WT was used as a reference. Values presented were mean +/− SD of 3 replicates of cDNA samples. **b** Growth phenotypes of WT, null lines, *CKX2*-overexpression heterozygous and homozygous lines. (a) Two-month-old plants. **b** Three-month-old plants. Agronomic traits analyzed upon harvest were (**c**) panicle number/plant and (**d**) total biomass/plant

In a separate experiment, we have obtained many transgenic rice lines overexpressing maize cytosolic carbonic anhydrase (CA) (Additional file 3: Figure S3A) and one of the lines exhibited reduced tiller number whereas other lines showed a normal tiller phenotype. Southern hybridization indicated the maize gene was inserted into the rice genome in a single copy (Additional file 3: Figure S3B). Using primers designed for the T-DNA LB and RB sequences we performed tail-PCR analysis and determined the insertion location of the gene on chromosome 1 at 5269546 bp or 98 bp of the 3′-UTR of *CKX2* (LOC_Os01g10110) (Additional file 3: Figure S3C). To further test the influence of this insertion on expression of *CKX2*, genotyping using primers based on the neighboring boarding sequences of the maize gene was used to determine the homozygosity among the T1 plants of this transgenic line. Among the T1 plants analyzed, two homozygous, 2 heterozygous and 2 null lines were selected for growth analysis in the greenhouse. As expected, western immunoblot analysis using specific antibodies against the maize CA showed that the homozygous plants expressed a high level of maize CA and the heterozygous plants expressed a lower level of the protein, while the WT and null lines did not express the maize protein at all (Additional file 4: Figure S4). As reported previously (Jeong et al. 2002; Wang 2008), insertion of a transgene under the control of a strong promoter in the 3′-UTR of another gene can lead to its activation, we found that the expression levels of *OsCKX2* in the T1 homozygous (Lines 1–6, 1–9) and heterozygous plants (1–28, 1–41) were about 13–13.5 and 3.5–6.5 fold higher than that of WT, respectively, whereas the null lines and WT had low levels of expression (Fig. 6a). High expression level of *CKX2* in the homozygous plants is expected to reduce cytokinin accumulation through increased oxidative degradation by the enzyme. Consistent with this hypothesis, agronomic trait analysis showed insertional activation or overexpression of *OsCKX2* led to reduced panicle number and total biomass in the heterozygous and homozygous plants, compared to WT and null lines (Fig. 6c, d). The homozygous plants showed low tiller numbers (7–11 tillers/plant), as compared to null lines (18–20) and WT (17–22 tillers/plant) (Fig. 6b). The heterozygous plants also exhibited reduced tiller numbers (13–15 tillers/plant) though not as low as homozygous plants. Among these T1 plants, the *OsCKX2* transcript levels were highly correlated with panicle number (*r* = 0.88) and total biomass (*r* = 0.89). Thus, our results revealed a clear gene-dosage effect on panicle number and biomass. To test if the changes in tiller number among these plants were related to the expression of rice *SPL14* (Luo et al. 2012), *D53* (Zhou et al. 2013) and *TB1* (Choi et al. 2012) that are known to be involved in regulation of tillering, we conducted qRT-PCR to examine the expression levels of these genes in the null, heterozygous and homozygous plants derived from the *OsCKX2* overexpression line. Our results showed clearly the expression of these genes was not significantly different from that of WT (Additional file 5: Figure S5A). Also, the expression levels of both rice *CKX1* and *CKX3* were similar to that of WT (Additional file 5: Figure S5B). Taken together, these results strongly support our hypothesis that insertional activation leads to increased expression and accumulation of *OsCKX2*, which in turn led to reduced tiller number/biomass, presumably due to a lower level of cytokinin accumulated in the shoots.

## Discussion

The goal of this study was to specifically down regulate the expression of rice *CKX2* using shRNA-mediated gene silencing to examine its influence on rice growth and productivity. Introduction of dsRNA- or shRNA-producing transgenes through transformation has been successively demonstrated in plants (Wang et al. 2000; Zalewski et al. 2010; Zhao et al. 2006), leading to a long-term gene silencing of the target mRNAs (Sliva and Schnierle 2010; Kim et al. 2012). Similarly, here we showed that *CX3*- or *CX5*-suppression transgenic rice lines expressed the shRNAs efficiently and specifically reduced the expression of homologous *OsCKX2* (Fig. 1b, Additional file 1: Figure S1B-C, Fig. 3) withouting affecting the expression of other *CKX* genes, such as *CKX1* and *CKX3* (Additional file 2; Figure S2). Our results are consistent with those of transgenic barley plants that harbored hairpin (hp) RNA-expression constructs containing barley yellow dwarf virus-PAV (BYDV-PAV) sequences (Wang et al. 2000) and partial cDNA fragments of *HvCKX1* (Zalewski et al. 2010), resulting in blockage of the translation of target mRNAs into proteins. Earlier studies also demonstrated that gene silencing by stable integration can be triggered by the presence of multiple transgene copies in transgenic plants with varying degrees of target reduction (Waterhouse et al. 2001; Kerschen et al. 2004). The *CX3*- or *CX5*-suppression transgenic rice lines in this study contained two or three copies of the silencing genes in the genome (Fig. 2), conferring an efficient reduction of *OsCKX2* transcript in most lines (Additional file 1: Figure S1B, 1C, Fig. 3). Our data also tend to suggest that *CX5* may be more efficient than *CX3* in suppression of *OsCKX2* expression (Fig. 3). Most importantly, the specific suppression of *OsCKX2* expression by *CX3*- or *CX5* was demonstrated in the progenies, suggesting that the silencing transgenes were stably inherited in the following generations. Kerschen et al. (2004) also reported a similar result on constitutive transcriptional transgene silencing in the T4 homozygous RNAi lines of *Arabidopsis*.

Cytokinins are known to play an important role in plant developmental and physiological processes (Mok 1994; Letham 1994). Consequently, they regulate crop yield by modifying the growth and development of both vegetative and reproductive tissues. An earlier study showed that exogenous application of cytokinins promotes chlorophyll accumulation in cucumber cotyledons (Fletcher and McCullagh 1971). Similarly, our *CX3*- and *CX5*-suppression rice lines also have elevated leaf chlorophyll contents at all stages (e.g. 3–9 % for 3-month-old plants), with a delayed leaf senescence (data not shown). This may be attributed to a higher cytokinin level in the plants, conferring the transgenic plants a higher photosynthetic capacity over a longer duration. In addition, an increase of endosperm cell number and grain weight of rice by treating roots, leaves and panicles with exogenous kinetin provides direct evidence for its role in regulating endosperm development (Yang et al. 2002). Consistently, maize kernels exposed to high temperature (35 °C) exhibit a decline in endogenous cytokinin levels, leading to disrupted endosperm development and starch biosynthesis (Cheikh and Jones 1994). Development of heat-stressed kernels can be recovered after treatment with exogenous BA (Benzyladenine) (Cheikh and Jones 1994). These results strongly suggest that development of reproductive organs is tightly regulated by cytokinins and high temperature inhibition of seed development is related to reduced cytokinins. Therefore, higher levels of cytokinins in the reproductive organs could lead to bigger or heavier grains, consistent with the observation in the present study (Fig. 5).

As CKX is the only known enzyme responsible for irreversible degradation of cytokinins in plants, mutant or transgenic plants expressing low levels of *CKX* genes have been shown to possess a clear cytokinin-overproducing phenotype. For example, rice cultivars with more grains in the panicle have a higher level of cytokinins in the inflorescence meristems (Ashikari et al. 2005). In *Arabidopsis*, a *CKX3*/*CKX5* double mutant forms more and larger flowers (Bartrina et al. 2011). Recent genetic studies through transformation suggest that *CKX* expression can be manipulated for enhanced crop productivity. In rice, decreased expression of *OsCKX2* in transgenic by antisense also leads to an increase in grain number in the panicles (Ashikari et al. 2005). There were more capsules produced in *CKX*-suppression tobacco plants caused by ihpRNA (intron-containing hairpin RNA) (Zeng et al. 2012). Also, downregulation of *CKX2* in both rice and barley by antisense and hpRNAi suppression resulted in enhanced grain yields (Ashikari et al. 2005; Zalewski et al. 2010). In the present study, the T3 homozygous *CX3-* and *CX5-*suppression lines produced significantly more tillers/panicles and grain yields on a per plant basis, and had a heavier 1000 grain weight, as compared to the wild type in both green house (Fig. 4) and field conditions (Fig. 5). Thus, our results suggest that specific down regulation of *OsCKX2* leads to enhanced tillering and thus a higher productivity in rice, presumably due to enhanced cytokinin levels. The higher chlorophyll contents and delayed leaf senescence in combination may have contributed to heavier grains.

A good understanding of the expression pattern of various *CKX* genes in different cultivars or in different organs of a given cultivar would be useful for identifying a specific target gene for manipulation of cytokinin contents. In this work, we showed that *OsCKX2* was highly expressed in the stem and inflorescence of TNG67, but negligible in the leaf and root (Additional file 1: Figure S1). The expression pattern of *OsCKX2* in TNG67 was generally consistent with another cultivar Taichung 65, except that a high level of *OsCK*X2 was detected in the leaf of the cultivar Taichung 65 (Ashikari et al. 2005). In contrast, barley *HvCKX1* transcript accumulates highly in developing spikes (0 to 14 DAP) and seedling roots (Zalewski et al. 2010); the expression pattern of other *HvCKXs* has not been characterized. Moreover, the expression of *OsCKX*s is subjected to cytokinin feed forward regulation. Multiple *OsCKX* genes are induced in response to cytokinin in rice root and/or in shoot, and *OsCKX2* is up-regulated in both root and shoot (Tsai et al. 2012). The maize *ZmCKX1* transcript accumulates in developing kernels (0 to 34 DAP), which is correlated with different levels of cytokinin oxidase activity and cytokinin contents (Brugiere et al. 2003). These results demonstrate the complexity in the expression and regulation of cytokinin degradation genes in plants and their importance as a key regulatory factor in crop productivity. Ashikari et al. (2005) suggested that the expression level of *OsCKX2* in different rice cultivars is inversely related to the content of cytokinins in inflorescence meristems, which in turn controls the rice grain number in main panicles. Although reduced levels of *OsCKX2* transcript were detected in the young stems of our *CX3*- and *CX5-*suppression plants, there were no significant increases in grain number in the panicles, except an increased tiller number and grain weight (Fig. 5). Consistently, our transgenic rice lines with insertional activation of *CKX2* had elevated expression of *CKX2* (Fig. 6a) and exhibited a reduced tiller phenotype and growth in a gene-dosage dependent manner (Fig. 6b). And the changes in tiller number are not related to the expression of three tiller related genes in rice, *SPL14*, *D53* and *TB1* (Additional file 5: Figure S5A). Thus, the results from both gain-of-function and loss-of-function experiments support our conclusion that specific down-regulation of cytokinin oxidase 2 expression enhances rice yield by increasing tiller number and grain weight. The lack of increased grain number by inhibiting *CKX2* expression, as observed in this study, may be caused by a lower expression of shRNAs in the inflorescence (i.e. a lower amount of cytokinins accumulated) to trigger differentiation of more florets in the panicles. Alternatively, the difference could be accounted for by the different cultivars used between the studies, as a certain QTL could be only applicable in certain varieties (Miura et al. 2011).

Taken together, our data demonstrated that the expression of the endogenous *OsCKX2* gene can be effectively suppressed by shRNA-*CX3* and -*CX5* in transgenic rice, resulting in increased chlorophyll content, growth, grain weight and yields. The increases in grain yield are mainly due to increased tillering capability and heavier grains. Most importantly, the cytokinin-overproducing phenotypes in the transgenic plants are stably inherited over the next generations. To the best of our knowledge, this is the first study in rice where efficient suppression of a specific target gene is successfully achieved by shRNAi. Thus, the shRNA-based gene silencing technique provides a simple new approach for manipulating rice productivity. Our results support the idea that manipulation of the *CKX* gene expression and the cytokinin level in crop plants can result in increased growth and productivity (Ashikari et al. 2005; Zalewski et al. 2010; Zeng et al. 2012). Furthermore, a cytokinin overproducing genotype may confer an adaptive significance at high temperatures, which is known to inhibit cytokinin accumulation in the seeds (Cheikh and Jones 1994).

## Conclusions

Rice is a staple food for more than half of the world population. To meet the expanding food demands for the rapidly growing world population, increase in rice yield and production is of utmost importance. Results from both gain-of-function and loss-of-function experiments in this study support the notion that specific down-regulation of cytokinin oxidase 2 expression enhances rice yield by increasing tiller number and grain weight. Thus, efficient suppression of *OsCKX2* expression through shRNA-mediated gene silencing provides a simple means to enhance rice growth and productivity.

## Methods

### Selection of siRNA Target Sites

The *N*-glycosylation sites of rice OsCKX2 (cytokinin oxidase/dehydrogenase; GenBank: AB205193) were identified using the PROSITE database (http://prosite.expasy.org/prosite.html) and NetNglyc 1.0 server (http://www.cbs.dtu.dk/services/NetNGlyc/). The analysis showed that *N*-glycosylation sites are present at amino acid residues 64–67 and 464–467 (Additional file 6: Table S1 and Additional file 7: Figure S6). Thus, nucleotides 182–202 and 1389–1409, which code for the *N*-glycosylation sites, were used for the construction of shRNAs as specific siRNAs against *OsCKX2* mRNA and designated *CX3* and *CX5*, respectively. Also, the 21 nt target sequences (*CX3*, *CX5*) were submitted to the NCBI/BLAST database to confirm their specificity for targeting *OsCKX2* mRNAs in rice.

### Construction of Transformation Vectors

**Fig. 7:**
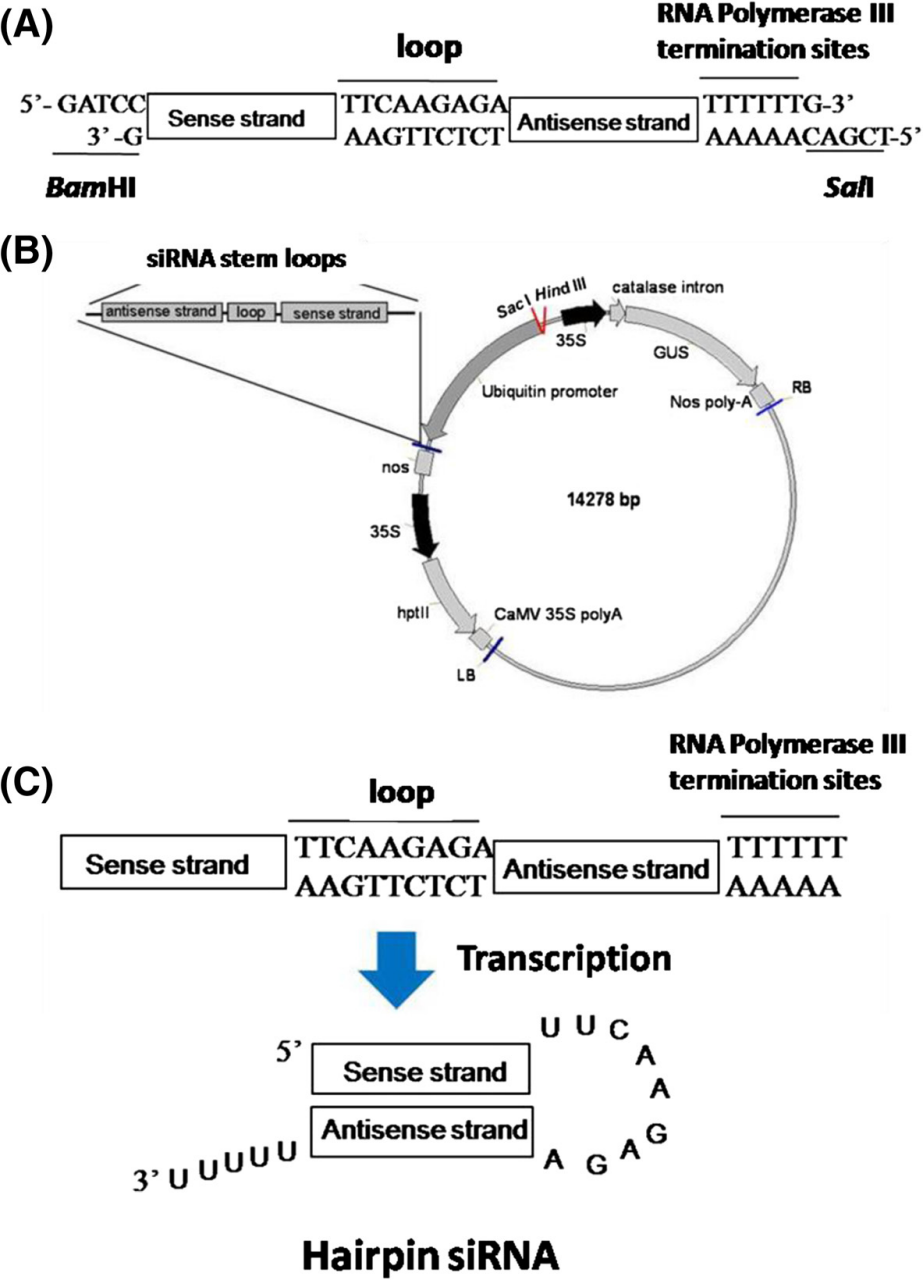
Construct of *p*C1301-*CX3* or -*CX5* containing a shRNA stem loop for rice transformation. **a** Diagram of a short hairpin RNA (shRNA) cassette. This cassette comprises inverted repeat sequences (*CX3* or *CX5*) against the target gene (*OsCKX2*), a spacer fragment (loop) and RNA polymerase III termination sites. **b** Schematic representation of the transformation constructs (not drawn to scale). shRNA stem loop: 21 nt sense and 21 nt antisense sequences linked by an unpaired loop sequence; Ubiquitin promoter: maize ubiquitin promoter; *35S*: CaMV 35S promoter; *nos*: nopaline synthase terminator; *GUS*: β-glucuronidase gene; *hptII*: hygromycin phosphotransferase II gene, LB: left border; RB: right border. **c** Diagram of self-complementary hairpin siRNA

To prepare vectors for shRNA-mediated gene silencing in rice, an oligonucleotide sequence (Additional file 8: Table S2) was designed for the loop linking sense and antisense strands, as described by Elbashir et al. (2001). shRNAs containing the loop sequence (TTCAAGAGA) have been shown to be highly efficient in silencing target genes (more than 90 %) (Brummelkamp et al. 2002). Thus, this loop sequence was adopted, while the siRNA target sequences (*CX3* and *CX5*) were designed in the sense and antisense orientations. Oligonucleotide sequences of *CX3*-Sense and -Antisense (or *CX5*-Sense and -Antisense) with *Bam*HI and *Sal*I restriction sequences at the 5′ and 3′ ends were synthesized and phosphorylated at the 5′ ends (Fig. 7).

To generate double-stranded DNA, equal molar concentration of both complementary oligos were mixed in an annealing buffer [10 mM Tris–HCl (pH 8.0), 50 mM NaCl, 1 mM EDTA], followed by heating at 94 °C for 5 min and slow cooling to room temperature for 45–60 min. After annealing, oligonucleotide sequences of *CX3*-Sense and *CX3*-Antisense (or *CX5*-Sense and *CX5*-Antisense) formed double-stranded DNAs with *Bam*HI and *Sal*I sites at the 5′ and 3′ ends, respectively (Fig. 7a). These vectors were designated shRNA-*CX3* and shRNA-*CX5*, accordingly.

shRNA-*CX3* or shRNA-*CX5* was subsequently cloned into a *Bam*HI-*Sal*I *p*Bluescribe M13+ vector to produce an intermediate *p*shRNA-*CX3* or -*CX5*. Positive clones were identified by sequencing; the *p*shRNA-*CX3* or -*CX5* vector was digested with *Xba*I, treated with Klenow polymerase to generate blunt ends, and ligated to a blunt-ended maize ubiquitin promoter fragment to produce *p*Ubi-*CX3* or -*CX5*. The orientation of the ubiquitin promoter in the positive clones was confirmed by restriction enzyme digestion. Finally, a *Sac*I-*Kpn*I shRNA-*CX3* or -*CX5* fragment carrying the ubiquitin promoter formed *p*Ubi-*CX3* or -*CX5*, and a *Kpn*I-*EcoR*I *nos* terminator fragment was ligated to a *Sac*I-*EcoR*I binary vector *p*CAMBIA1301 to produce the final plasmid *p*C1301-*CX3* or *p*C1301-*CX5* (Fig. 7c). The final constructs were transfected via electroporation into the *Agrobacterium tumefaciens* strain AGL1.

### Expression of siRNA with Stem Loop Structure in Transgenic Rice

The *p*C1301-*CX3* or *p*C1301-*CX5* vector containing shRNA-*CX3* or -*CX5* with loop sequences was used to silence homologous *OsCKX2* expression in rice after transformation. This shRNA stem loop structure with target DNA sequences of *CX3* (ACTTTGGCAACCTCTCCGTCGTTCAAGAGACGACGGAGAGGTTGCCAAAGT) or *CX5* (TAACATGTCGGCAGTGATCACTTCAAGAGAGTGATCACTGCCGACATGTTA) was inserted in the sense-loop-antisense orientation under the control of the maize ubiquitin promoter (Fig. 7b). The underlined nucleotides represent the loop sequence.

The basic transcriptional unit of hairpin siRNA was produced as follows. The inverted repeat sequences of *CX3* or *CX5* (also called the stem) and a spacer loop formed a self-complementary hairpin structure after transcription in plants (Fig. 7c). The stem region of hairpin RNAs was then processed into 21-nt siRNAs by RNase III enzymes Dicer (Sliva and Schnierle 2010; Kim et al. 2012). In addition, the *p*C1301-*CX3* or *p*C1301-*CX5* vector contained β-glucuronidase (*GUS*) and hygromycin phosphotransferase II (*hpt*II) genes driven by CaMV 35S promoters (Fig. 7b). These two genes were used as selectable markers for screening primary transformants.

### Rice Transformation and Regeneration

Rice transformation through *A. tumefaciens* was carried out according to the methods described previously (Ku et al. 1999; Toki 1997). Calli induced from immature seeds of rice (*Oryza sativa* cv. TNG67) were used for rice transformation. The yellow compact embryogenic calli were co-cultured with *A. tumefaciens* strain AGL1 carrying the plasmid *p*C1301-*CX3* or *p*C1301-*CX5* and incubated at 28 °C in dark for 3–4 days. After co-cultivation, calli were thoroughly washed with 250 mg L^−1^ cefotaxime in sterile distilled water and transferred to a selection medium containing 50 mg L^−1^ hygromycin B (Invitrogen) and 250 mg L^−1^ cefotaxime at 28 °C in light for one month. Healthy hygromycin-resistant calli were subsequently transferred to a MS regeneration medium supplemented with 2,5 mg L^−1^ kinetin, 1 mg L^−1^ NAA and 50 mg L^−1^ hygromycin. Transgenic plantlets were transferred to a hormone-free 1/2 MS medium without hygromycin in magenta boxes for 10–14 days to promote root growth. Calli selection and plant regeneration were conducted in a growth chamber at 28 °C and 60 % relative humidity under a 16 h/8 h light/dark photoperiod. Transgenic seedlings were transplanted in soil, cultured in the greenhouse, and self-pollinated for seed production.

### PCR, Semi-Quantitative RT-PCR (RT-PCR) and Quantitative Real-Time PCR (qRT-PCR) Analyses

To detect transgenes and to evaluate the *OsCKX2* transcript level in transgenic lines, specific primers were used in PCR, RT-PCR and qRT-PCR analyses (Table 1). For PCR analysis, genomic DNA was isolated from leaves of transgenic and WT (wild type) plants, as described by Sheu et al. (1996). Amplification of *GUS* and *CX3* or *CX5* transgenes was performed in a thermal cycler under the following conditions: 94 °C/5 min, 30 cycles of 94 °C/1 min, 56 °C/1 min and 72 °C/1 min, and a final extension at 72 °C/5 min. The conditions for amplification of *hptII* were 94 °C/5 min, 28 cycles of 94 °C/1 min, 65 °C/1 min and 72 °C/1 min, and a final extension at 72 °C/5 min.

**Table 1:** Specific primers used in this study

Primer name	Sequence (5′ to 3′)	Target gene
Hygromycin-F	GACCTGATGCAGCTCTCGGAG	*hptII*
Hygromycin-R	TGCTCCATACAAGCCAACCACG	
GUS-F	AAAAAACTCGACGGCCTGTGGG	*GUS*
GUS-R	GCATCTTCATGACGACCAAAGC	
Ubiquitin pro.-F	CTGATGCATATACAGAGATGC	^a^ *CX3* or *CX5*
Nos ter.-R	TGACAGCTTATCATCGGATC	
CKX2-F	GTCCACGACGGCGAGCTCAA	*OsCKX2*
CKX2-R	TCCATCTTGGCATCTCTCAG	
Actin-F	GGTAATGTGTTGGACTCTGG	*Actin*
Actin-R	GCAGTGATCTTCCTTGCTCA	
3′UTR-F	CGGTGACGAGGTGTTCTACAC	*OsCKX2* 3′ UTR
3′UTR -R	CCAAGATCTCGTCGTTCTGC	
rRNA-F	GATAACTCGACGGATCGCACGG	*17S rRNA*
rRNA-R	GCCTGCTGCCTTCCTTGGATGTG	
OsCKX1-F	GCACCCGTGGCTCAACCTG	*CKX1*
OsCKX1-R	GATGTCGGTGGCCGTCTGG	
OsCKX3-F	TTTCTTATGCTGATGTGGGTG	*CKX3*
OsCKX3-R	GAACATTGCTAATCTGAGGTCC	
OsSPL14-F	TGAATTTGACCAAGGAAAAC	*SPL14*
OsSPL14-R	ATCCAACGTAAAGCTTCTGA	
D53-F	CCAAGCAGTTTGAAGCGAC	*D53*
D53-R	CCGCAAGTTTATCAAAGTCAA	
OsTB1-F	CAAGAAATCTCGGCGGCTAG	*TB1*
OsTB1-R	CGAATTGGCGTAGACGAC	

^a^shRNA cannot be amplified directly by PCR. Thus, a 867 bp fragment containing partial promoter, shRNA and partial terminator were amplified for detecting the *CX3* or *CX5* gene

For RT-PCR and qRT-PCR analyses, total RNA was isolated from different tissues, as described by Wang and Vodkin (1994). Total RNA was first treated with RNase-free DNase I to remove genomic DNA and the resulting RNA was used for first-strand cDNA synthesis with an Oligo (dT) primer and M-MLV reverse transcriptase (Promega) (Chen et al. 2006). Two μg cDNA was used for gene expression assay by RT-PCR, conducted at 94 °C/5 min, 28 cycles of 94 °C/1 min, 58 °C/1 min and 72 °C/1 min, and a final extension at 72 °C/5 min. For qRT-PCR, the protocols of Livak and Schmittgen (2001) and Yeh et al. (2014) were followed using 3′URT specific primers for *OsCKX2* (Table 1). Expression of *17S rRNA* (accession number X00755) (Table 1) was used as an internal control for normalization of expression levels.

### Southern Blot Analysis

Southern gel blot analysis was performed according to the method of Ku et al. (1999). Genomic DNA was digested with *Sac*I or *Hin*dIII, electrophoresed on 1 % agarose gel and transferred to a nylon membrane for probe hybridization. The *hptII*, *CX3*, and *CX5* fragments, either amplified by PCR or excised from the plasmids *p*C1301-*CX3* and *p*C1301-*CX5* by restriction enzyme digestion, were used as probes. Probes were radioactively labeled via random primer labeling, and hybridization was performed at 65 °C for 8–12 h. After hybridization membrane was stripped off non-specific probes successively in washing solution containing 2× SSC/0.1 % SDS and then in solution containing 0.1× SSC/0.1 % SDS at 45 °C for 10–20 min.

### Northern Blot Analysis

Total RNA was isolated from different organs, as described by Wang and Vodkin (1994). RNA gel blot analysis was performed according to the method described by Chen et al. (2006). Total RNA was denatured with formaldehyde and formamide, electrophoresed on 1.2 % agarose gel and transferred to nylon membrane. Hybridization probes were radioactively labeled using a random primer labeling method. Hybridization and washing procedures were the same as described in Southern analysis.

### Antibodies and Western Immunoblotting

Antibodies against the maize carbonic anhydrase (CA) were produced against the synthetic peptide (KKKEGPAKEKPSTDTP), specific to the maize protein, in rabbits and purified by affinity chromatography (Yao-Hong Biotechnology Inc., Taiwan). The protocol of Dai et al. (1994) was used for SDS-PAGE and western immunodetection.

### Field Experiments

Field tests were carried out in the transgenic field of Taiwan Agricultural Research Institute in random block design, with each block containing 60 transgenic or WT seedlings (1 seedling/hill). Row spacing was 30 cm and plant spacing was 15 cm. For each line, after excluding the boarding plants 5 randomly chosen plants were harvested and pooled as one replicate and a total of 8 replicates of plant samples were harvested for analysis. Total biomass, panicle number, total grain weight and total grain number per plant, and 1000 grain weight were analyzed after drying (43 °C/2 days for seeds and 60 °C/2 days for stems and leaves). Data presented were mean ± SD with a significance level at *P* < 0.05.

## Additional files

Additional file 1: Figure S1. Expression of *OsCKX2* transcript in different rice organs of untransfomed wild type (WT) and in the young stems of *CX3*- and *CX5*-suppression lines, as assayed by RT-PCR (A, B) and by northern blot analysis (C). (A) Total RNA was isolated from leaf (L), root (R), stem (S), inflorescence (I), and green floret (F) of untransformed WT. (B) Total RNA was isolated from the young stems of untransformed WT and selected T1 transgenic lines. Expression of *actin* was included as a cDNA loading control. (C) RNA gel blot analysis of *OsCKX2* transcript in the young stems of untransformed WT and selected T1 transgenic lines. Total RNAs isolated from transgenic lines harboring shRNA-*CX3* (C-a), and shRNA-*CX5* (C-b) were hybridized with a ^32^P-labelled *OsCKX2* probe. Ethidium bromide staining of rRNA indicated an equal loading of RNA in each sample. (DOC 241 kb) Additional file 2: Figure S2. Relative expression levels of rice *CKX1* and *CKX3* in WT and selected T1 *CX3*- and *CX5*-suppression lines, as assayed by qRT-PCR. Total RNA was isolated from the young stems of wild type (WT) and selected T1 *CX3*-suppression lines (3–5–2, 3–5–7) and *CX5*-suppression lines (1–2–2, 1–2–4). Water (NC) was included as a negative control and *17S rRNA* was used as an internal control for normalization. The expression level of WT was used as a reference. Values presented were mean +/− SD of 3 replicates of cDNA samples. (DOC 70 kb) Additional file 3: Figure S3. (A) *p*CAMBIA-1300 based construct used for overexpressing maize cytosolic carbonic anhydrase (*CA*) gene in rice. Maize PEPC gene promoter was used to drive the expression of *CA* and 35S prmomter driven *bar*, which encodes DPAT (demethylphosphinothricin acetyltransferase), was used for selection of transgenic plants by the herbicide bialophos. (B) Southern blot analysis of *bar* in wild type (WT) and selected T0 *CKX2*-overexpressiom transgenic lines (1, 2, 3, 5, 9, 10) containing the maize carbonic anhydrase gene. Plasmid DNA (P) was used as a positive control. Genomic DNA was digested with *Hin*dIII, resolved by electrophoresis and hybridized with DIG labeled *bar* probe. (C) Location of insert fragment on rice chromosome 1. (DOC 108 kb) Additional file 4: Figure S4. Western blot analysis of carbonic anhydrase (49.5 kDa) in wild type (WT) and T1 null (1–4, 1–18) and *CKX2*-overexpression heterozygous (1–28, 1–41) and homozygous (1–6, 1–9) lines. Maize was used as a positive control. (DOC 61 kb) Additional file 5: Figure S5. Relative expression levels of three rice tiller related genes, *SPL14*, *D53* and *TB1* (A), and *CKX1* and *CKX3* (B) in representative T2 *CKX2*-overexpression lines by qRT-PCR. Total RNA was isolated from the shoot apex of wild type (WT) and null lines (1–4–1, 1–18–1), T2 *CKX2*-overexpression heterozygous (1–28–16, 1–41–6) and homozygous (1–6–2, 1–9–5) lines. *17S rRNA* was used as an internal control for normalization. The expression level of WT was used as a reference. Values presented were mean +/− SD of 3 replicates of cDNA samples. (DOC 146 kb) Additional file 6: Table S1. Significant putative sites present in the amino acid sequence of OsCKX2. (DOC 30 kb) Additional file 7: Figure S6. Predicted N-glycosylation sites in the amino acid sequence of OsCKX2. (DOC 52 kb) Additional file 8: Table S2. Oligonucleotides used in the study. (DOC 27 kb)

## Abbreviations

CA: carbonic anhydrase
CKX: cytokinin oxidase/dehydrogenase
GUS: β-glucuronidase
hpRNAi: hairpin RNA interference
hptII: hygromycin phosphotransferase II
qRT-PCR: quantitative real-time PCR
QTLs: quantitative trait loci
RT-PCR: semi-quantitative PCR
shRNA: short hairpin RNA
