# Supplementary figures and images for: Down-Regulation of Cytokinin Oxidase 2 Expression Increases Tiller Number and Improves Rice Yield
Source: Rice (N Y). 2015 Dec 7;8:36. doi: 10.1186/s12284-015-0070-5 (PMC4671980; doi:10.1186/s12284-015-0070-5)

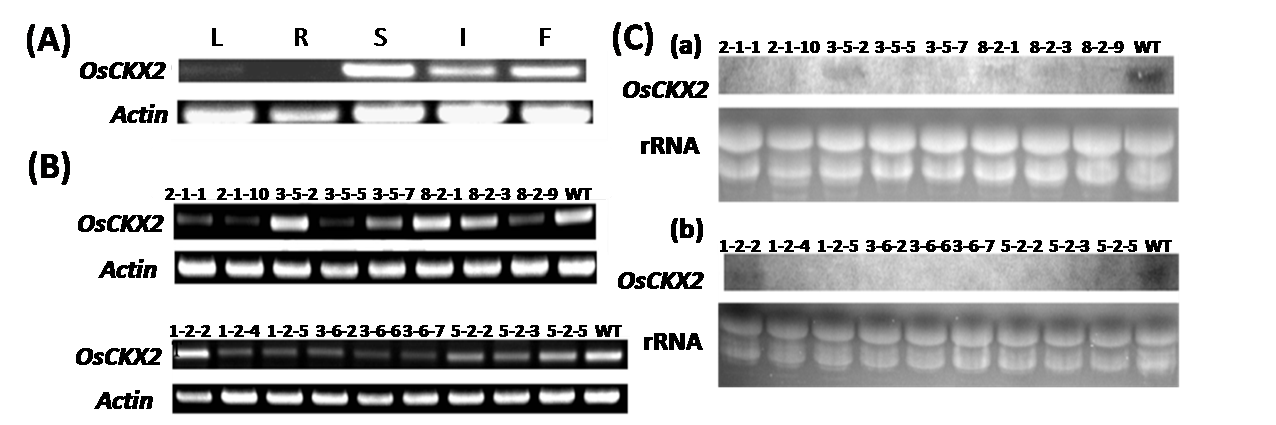


**Additional file 1: Figure S1.**

Supplement: Additional file 1: Figure S1. — Expression of OsCKX2 transcript in different rice organs of untransfomed wild type (WT) and in the young stems of CX3- and CX5-suppression lines, as assayed by RT-PCR (A, B) and by northern blot analysis (C). (A) Total RNA was isolated from leaf (L), root (R), stem (S), inflorescence (I), and green floret (F) of untransformed WT. (B) Total RNA was isolated from the young stems of untransformed WT and selected T1 transgenic lines. Expression of actin was included as a cDNA loading control. (C) RNA gel blot analysis of OsCKX2 transcript in the young stems of untransformed WT and selected T1 transgenic lines. Total RNAs isolated from transgenic lines harboring shRNA-CX3 (C-a), and shRNA-CX5 (C-b) were hybridized with a 32P-labelled OsCKX2 probe. Ethidium bromide staining of rRNA indicated an equal loading of RNA in each sample. (DOC 241 kb) [file 12284_2015_70_MOESM1_ESM.doc]

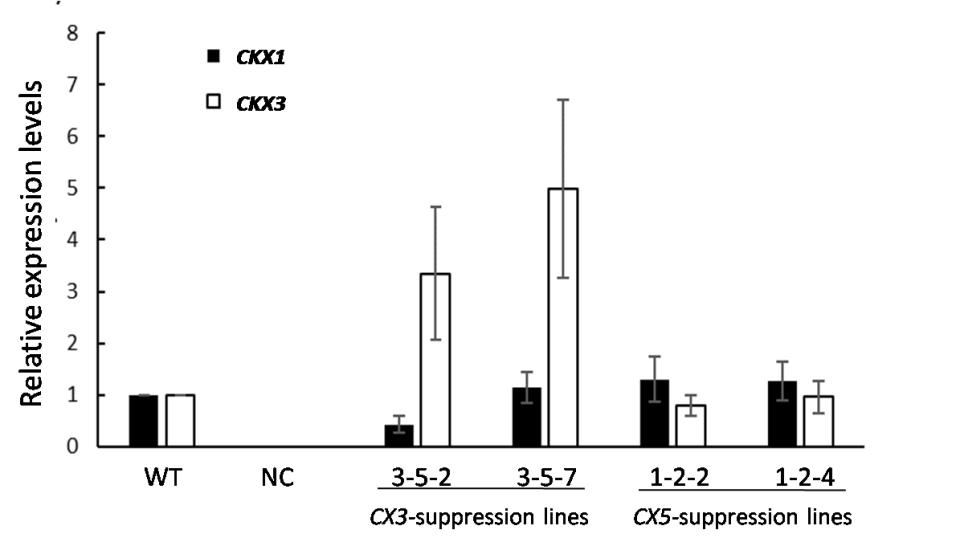


**Additional file 2: Figure S2.**

Supplement: Additional file 2: Figure S2. — Relative expression levels of rice CKX1 and CKX3 in WT and selected T1 CX3- and CX5-suppression lines, as assayed by qRT-PCR. Total RNA was isolated from the young stems of wild type (WT) and selected T1 CX3-suppression lines (3–5–2, 3–5–7) and CX5-suppression lines (1–2–2, 1–2–4). Water (NC) was included as a negative control and 17S rRNA was used as an internal control for normalization. The expression level of WT was used as a reference. Values presented were mean +/− SD of 3 replicates of cDNA samples. (DOC 70 kb) [file 12284_2015_70_MOESM2_ESM.doc]

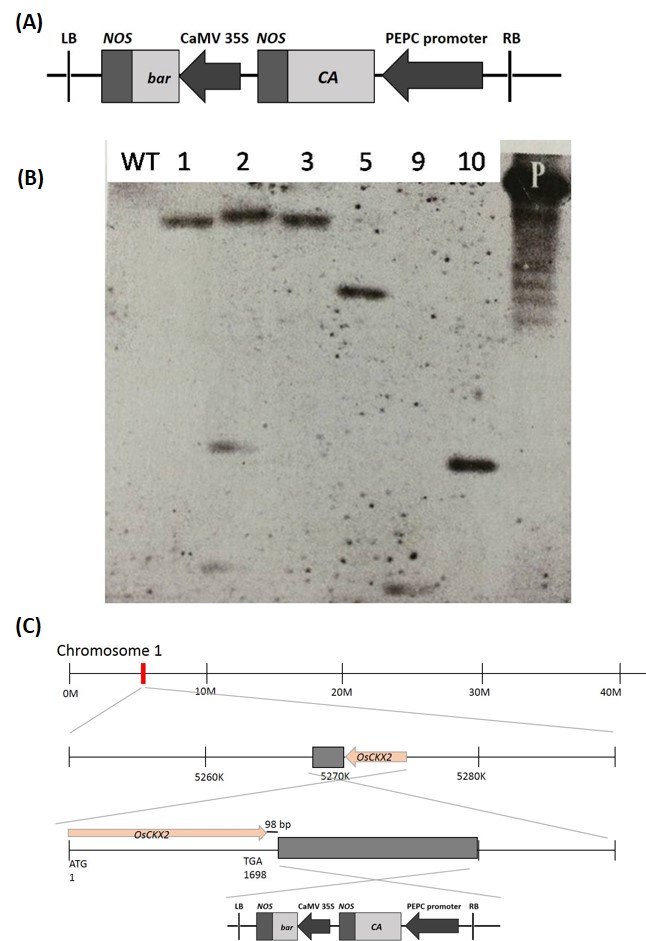


**Additional file 3: Figure S3.**

Supplement: Additional file 3: Figure S3. — (A) pCAMBIA-1300 based construct used for overexpressing maize cytosolic carbonic anhydrase (CA) gene in rice. Maize PEPC gene promoter was used to drive the expression of CA and 35S prmomter driven bar, which encodes DPAT (demethylphosphinothricin acetyltransferase), was used for selection of transgenic plants by the herbicide bialophos. (B) Southern blot analysis of bar in wild type (WT) and selected T0 CKX2-overexpressiom transgenic lines (1, 2, 3, 5, 9, 10) containing the maize carbonic anhydrase gene. Plasmid DNA (P) was used as a positive control. Genomic DNA was digested with HindIII, resolved by electrophoresis and hybridized with DIG labeled bar probe. (C) Location of insert fragment on rice chromosome 1. (DOC 108 kb) [file 12284_2015_70_MOESM3_ESM.doc]

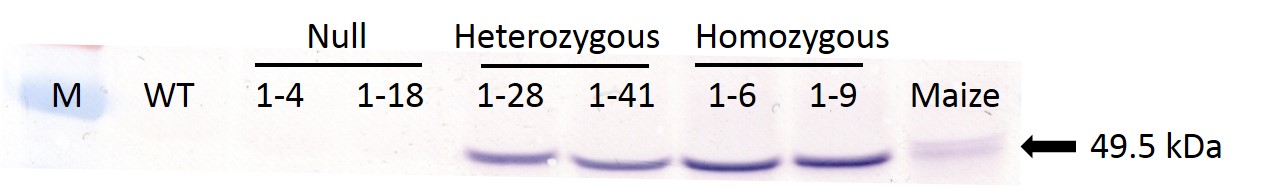


**Additional file 4: Figure S4.**

Supplement: Additional file 4: Figure S4. — Western blot analysis of carbonic anhydrase (49.5 kDa) in wild type (WT) and T1 null (1–4, 1–18) and CKX2-overexpression heterozygous (1–28, 1–41) and homozygous (1–6, 1–9) lines. Maize was used as a positive control. (DOC 61 kb) [file 12284_2015_70_MOESM4_ESM.doc]

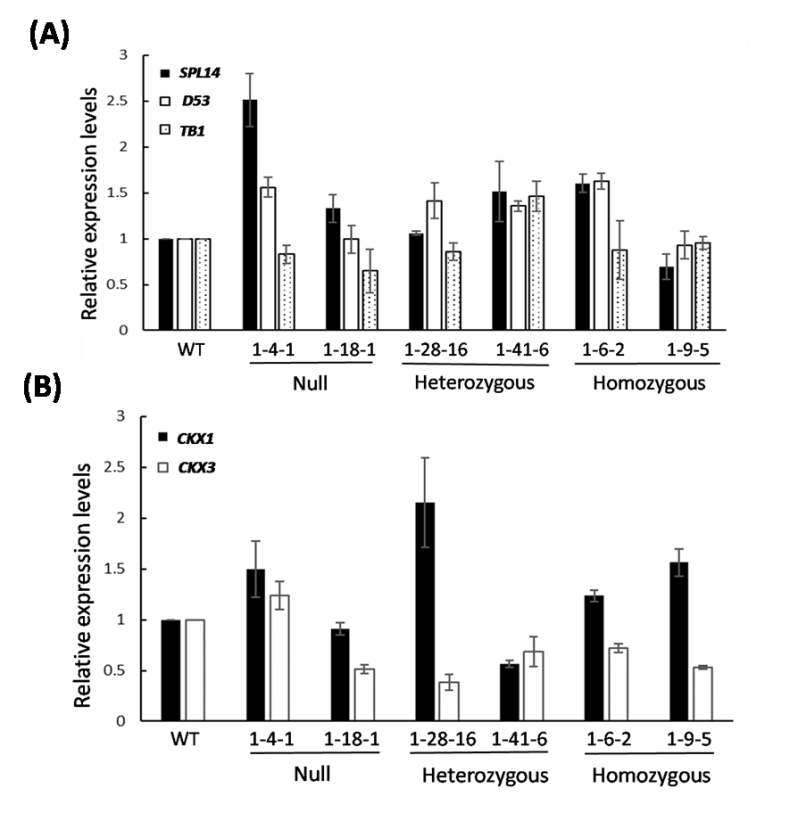


**Additional file 5: Figure S5.**

Supplement: Additional file 5: Figure S5. — Relative expression levels of three rice tiller related genes, SPL14, D53 and TB1 (A), and CKX1 and CKX3 (B) in representative T2 CKX2-overexpression lines by qRT-PCR. Total RNA was isolated from the shoot apex of wild type (WT) and null lines (1–4–1, 1–18–1), T2 CKX2-overexpression heterozygous (1–28–16, 1–41–6) and homozygous (1–6–2, 1–9–5) lines. 17S rRNA was used as an internal control for normalization. The expression level of WT was used as a reference. Values presented were mean +/− SD of 3 replicates of cDNA samples. (DOC 146 kb) [file 12284_2015_70_MOESM5_ESM.doc]

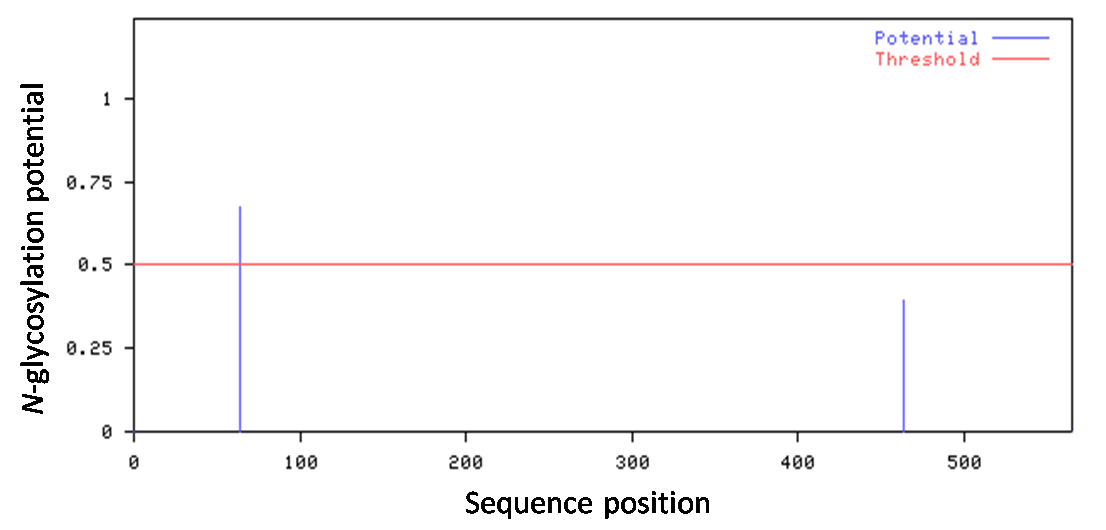


**Additional file 7: Figure S6.**

Supplement: Additional file 7: Figure S6. — Predicted N-glycosylation sites in the amino acid sequence of OsCKX2. (DOC 52 kb) [file 12284_2015_70_MOESM7_ESM.doc]
