# Supplementary material for: Down-Regulation of Cytokinin Oxidase 2 Expression Increases Tiller Number and Improves Rice Yield
Source: Rice (N Y). 2015 Dec 7;8:36. doi: 10.1186/s12284-015-0070-5 (PMC4671980; doi:10.1186/s12284-015-0070-5)
Supplement: Additional file 6: Table S1. — Significant putative sites present in the amino acid sequence of OsCKX2. (DOC 30 kb) [file 12284_2015_70_MOESM6_ESM.doc]

**Additional file 6: Table S1.**

| **Significant putative sites** | **Location** | **Amino acid sequence** |
| --- | --- | --- |
| Protein kinase C phosphorylation sites | 55-57 | TaR |
|  | 107-109 | SaR |
|  | 311-313 | TwR |
| *N*-glycosylation sites | 64-67 | NLSV |
|  | 464-467 | NMSA |
| FAD binding domain | 89-241 |  |
| FAD/FMN-containing dehydrogenases | 89-559 |  |
