# Supplementary material for: Down-Regulation of Cytokinin Oxidase 2 Expression Increases Tiller Number and Improves Rice Yield
Source: Rice (N Y). 2015 Dec 7;8:36. doi: 10.1186/s12284-015-0070-5 (PMC4671980; doi:10.1186/s12284-015-0070-5)
Supplement: Additional file 8: Table S2. — Oligonucleotides used in the study. (DOC 27 kb) [file 12284_2015_70_MOESM8_ESM.doc]

**Additional file 8: Table S2.**

| **Name**  **Oligonucleotide sequence (5'→3')** |
| --- |
| ***CX3*-Sense** GATCCACTTTGGCAACCTCTCCGTCGTTCAAGAGACGACGGAGAGGTTGCCAAAGTTTTTTG ***CX3*-Antisense** TCGACAAAAAACTTTGGCAACCTCTCCGTCGTCTCTTGAACGACGGAGAGGTTGCCAAAGTG ***CX5*-Sense** GATCCTAACATGTCGGCAGTGATCACTTCAAGAGAGTGATCACTGCCGACATGTTATTTTTG ***CX5*-Antisense:** TCGACAAAAATAACATGTCGGCAGTGATCACTCTCTTGAAGTGATCACTGCCGACATGTTAG |
